# TGF-β changes cyto/mito-ribosome balance to target respiratory chain complex V biogenesis in pulmonary fibrosis therapy

**DOI:** 10.1038/s41392-023-01370-2

**Published:** 2023-03-21

**Authors:** Na Zhang, Zunling Zhao, Yin Zhao, Lei Yang, Yanhong Xue, Yun Feng, Jianjun Luo, Runsheng Chen, Wei Wei, Yan Qin

**Affiliations:** 1 Department of Rheumatology and Immunology, Tianjin Medical University General Hospital, 300052 Tianjin, China; 2 CAS Center for Excellence in Biomacromolecules, Institute of Biophysics, Chinese Academy of Sciences, 15 Datun Road, Chaoyang District, 100101 Beijing, China; 3 University of Chinese Academy of Sciences, 100049 Beijing, China

**Keywords:** Cell biology, Respiratory tract diseases

**Dear Editor**,

Pulmonary fibrosis (PF) is characterized by failed alveolar re-epithelialization and fibroblast activation.^1^ A continuing struggle in the field has been how to diagnose and treat the disease early and effectively on the basis of shared pathogenetic mechanisms. Transforming growth factor-β (TGF-β) signaling and mitochondria are involved in PF pathogenesis.^2^ Mitochondrial respiratory chain complexes (RCCs) I, III, IV, and V comprise both nuclear DNA (nDNA)- and mitochondrial DNA (mtDNA)-encoded subunits, and their biogenesis depends on the cooperation of cytoplasmic (cyto) and mitochondrial (mito) translation (Supplementary Fig. 1).^3^ To date, a correlation between PF and RCC biogenesis has not been reported.

We first examined the TGF-β levels and the enzymatic activity of five RCCs in blood samples of healthy controls (HCs) and patients with the two most common forms of PF,^4^ idiopathic PF (IPF) and connective tissue disease PF (CTD-PF). Decreased RCCs activities, especially complex V, were observed in PF patients. Decreased complex V activity was significantly correlated with increased expression of TGF-β. Further receiver-operating characteristic (ROC) analysis revealed that the PF group and the HC group were well distinguished based on complex V activity levels and especially based on the combination of complex V activity and serum TGF-β levels. In addition, the extent of the fibrotic pattern on high-resolution computed tomography (HRCT) scans was correlated with complex V activity. These data suggest a potential role for the TGF-β +/complex V− combination as a PF blood biomarker (Fig. 1a–f, Supplementary Fig. 2, and Supplementary Table 1). Data from a bleomycin (BLM)-induced PF mouse model supported the above results, quantitatively and qualitatively indicating increased expression of TGF-β as well as decreased activity of complex V in both pulmonary tissue and blood samples. Besides, correlation analyses also reflected that there was the correlation between TGF-β, complex V activity and fibrotic lesions (Fig. 1g, Supplementary Figs. 3, S4, and Supplementary Tables 2 and 3).

**Fig. 1:**
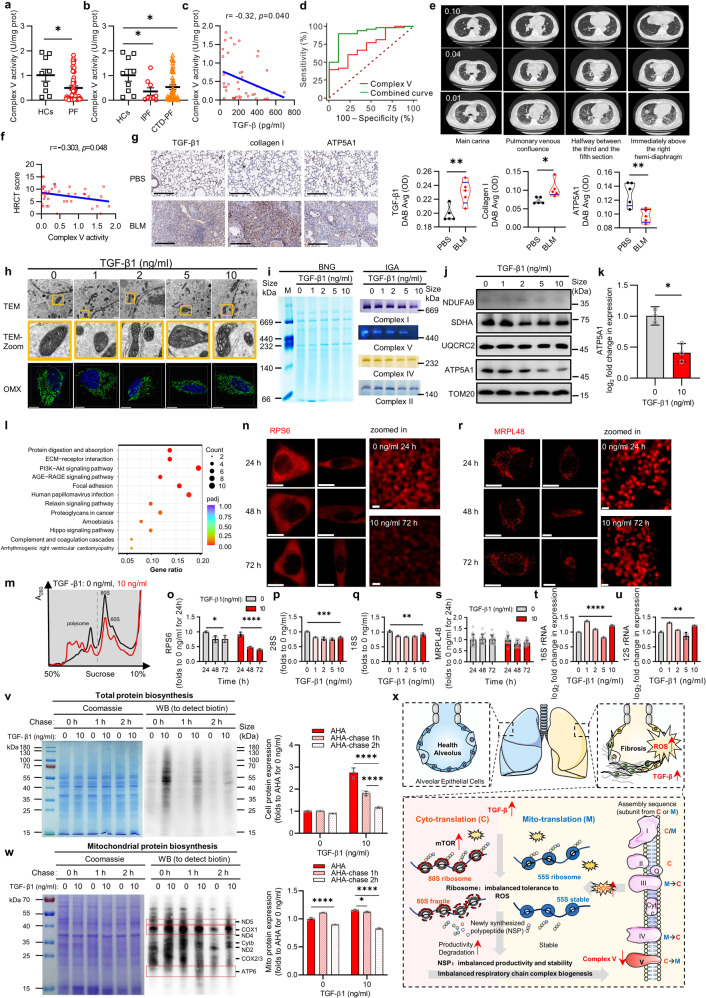
Respiratory chain complex V reduction indicates cyto/mito-ribosome imbalance and their different tolerances to free radical bursts in pulmonary fibrosis. **a** Complex V activity of pulmonary fibrosis patients (PF) (*n* = 50) and healthy controls (HCs) (*n* = 9). **b** Complex V activity of idiopathic PF (IPF) (*n* = 41), and connective tissue disease PF (CTD-PF) (*n* = 9) and HCs (*n* = 9). **c** Correlation analysis of complex V activity and serum TGF-β levels. **d** Receiver-operating characteristic (ROC) curve analysis of complex V activity (red)/ combined complex V activity and serum TGF-β levels (green) between PF patients (*n* = 50) and HCs (*n* = 9). **e** High-resolution computed tomography (HRCT) images at four sections of three patients with different complex V activity levels (0.10, 0.04, and 0.01). **f** Correlation analysis between complex V activity and HRCT score of PF patients (*n* = 50). **g** Immunohistochemical analysis and quantification of TGF- β1, collagen I and ATP5A1 in lung tissue of mouse model (*n* = 5). **h** A549 cells were treated with different concentrations of TGF-β1 for 48 hours, and images were taken using transmission electron microscope (TEM) and OMX mode of structural light illumination microscope, respectively. TEM images showed the changes of mitochondrial cristae under different experimental conditions, the images were taken using Tecnai Spirit (120 kV), scale bar = 500 nm. The OMX images showed the 3D changes of mitochondrial (green) morphology under different treatment conditions, the images were taken using SIMOMX with a ×60 oil-immersion objective, scale bar = 10 µm. **i** Blue native gel (BNG) and in gel activity (IGA) of mitochondrial respiratory chain complex I, complex II, complex IV, complex V of purified mitochondria. **j** Immunoblot for protein expression levels of NDUFA9, SDHA, UQCRC2, ATP5A1, and TOM20 in HLF cells lysates with different treatment conditions. All lanes were loaded with the same amount of total protein. **k** Quantification of ATP5A1 in ACEII cells with TGF-β1 treatments. **l** Annotation of transcripts of differential expression of genes in A549 cells induced by TGF-β1 at 0 and 10 ng/ml in KEGG database (*n* = 3). **m** The polysome difference between the 10 ng/ml treatment group and the 0 ng/ml control group. **n**, **r** The expression of RPS6 (**n**) and MRPL48 (**r**) in cells was detected by immunohistochemistry. The images were taken using Nikon STED with a ×100 oil-immersion objective under the same parameter settings, scale bar = 10 μm, zoomed in: scale bar = 200 nm. **o**, **s** Fold changes of RPS6 (**o**, *n* = 3) and MRPL48 (**s**, *n* = 15) expression in A549 cells induced by TGF-β1, the mean fluorescence intensity was obtained by Image J for statistics. **p**, **q** Fold changes of total RNA detected by agarose gel electrophoresis in A549 cells induced by TGF-β1, statistics were made by the grayscale value of electrophoretic band strips measured by Image J. **t**, **u** Fluorescence quantitative PCR analysis of mitochondrial 16 S and 12 S (*n* = 3). **v**, **w** L-AHA (a homolog of L-Met) detection of the ribosome translational function of living cells and mitochondria, statistics were made by the grayscale value of western blot strips measured by Image J (*n* = 3). **x** Respiratory chain complex V reduction indicates cyto-/mito-translation imbalance and different tolerances to free radical burst in pulmonary fibrosis. One-way ANOVA with Tukey’s multiple comparisons test and student *t* test were performed. *P < 0.05; **P < 0.01; ***P < 0.001; ****P < 0.0001

The human alveolar epithelial cell line A549, the epithelial-like cell line H1299, the bronchial epithelial cell line 16HBE, human lung fibroblasts (HLFs), and mouse primary type II alveolar epithelial cells (AECII) were used to explore the cellular details and molecular mechanism. As the concentration of TGF-β increased, more cells exhibited a spindle-shaped mesenchymal cell morphology similar to that of fibroblasts. Under transmission electron microscopy (TEM), the mitochondria displayed a swollen morphology and disordered cristae structure. Reduced numbers of cristae, vacuolization and slight staining in the bottom edge regions (where complex V is located) suggested a critical complex V defect (Fig. 1h, Supplementary Fig. 5, and Supplementary Video 1). To examine each complex quantitatively and qualitatively, blue native gel (BNG), in-gel activity (IGA) assays and western blotting (WB) were performed. In general, all complexes displayed decreases in both amount and activity as the TGF-β concentration increased. However, complex V decreased first and most significantly. Adenosine triphosphate (ATP) production also decreased, but a reactive oxygen species (ROS) burst occurred (Fig. 1i–k and Supplementary Figs. 6 and 7). Analysis of transcriptomic sequencing data with the Kyoto Encyclopedia of Genes and Genomes (KEGG) showed that the phosphatidylinositol 3-kinase (PI3K)-mammalian target of rapamycin (mTOR) pathway was the most highly enriched pathway, and translation enhancement was observed in the increased portion of the polysomes in ribosomal profiling. However, the total amount of ribosomes decreased because the amounts of monomers plummeted (Fig. 1l, m and Supplementary Fig. 8).

Considering that the cytoribosome (80 S) has an RNA outer layer but that the mito-ribosome (55 S) is covered by a tight protein enclosure that protects it from the frequently changing redox state in the mitochondrial matrix, we hypothesized that there were differences in the tolerance of the two ribosomes to a TGF-β + /complex V− state and subsequent intracellular stress. Cyto-80S protein S6 and mito-55S protein L48 were stained, and confocal microscopy and superresolution-stimulated emission depletion (STED) microscopy with SVl Htygens deconvolution to reach a 2-dimensional resolution of approximately 30 nm showed decreased 80 S levels but stable 55 S levels (Fig. 1n–u and Supplementary Fig. 9).

The de novo protein biosynthesis and product stability of both the cyto- and mito-translation systems were examined by click-iT l-azidohomoalanine (AHA) and chase assays. Cyto-translation exhibited enhanced productivity, in agreement with the PI3K-mTOR upregulation data above, but a very fast turnover rate and low stability of the protein products were observed. In contrast, mito-translation in both untreated and TGF-β-treated samples exhibited constant values for all 13 mtDNA-encoded polypeptides. More interestingly, the stability of ATP6, a complex V subunit, differed from that of the other complexes. Compared to the ND5 band, which had a similar expression level as ATP6 under TGF-β stimulation after 1 hour of chase, the ATP6 band was nearly undetectable after 2 h of chase (Fig. 1v, w and Supplementary Fig. 10). These results were consistent with our findings that TGF-β signaling enhanced cyto-translation but ultimately damaged cyto-ribosomes. The consequence of such cyto-translation was a quantitative increase in protein products but a decrease in the quality of the newly synthesized polypeptides, which might have been of normal length but lacked complete folding or maturation and prompt degradation. In contrast, the mito-ribosomes were much more stable, and the mtDNA-encoded respiratory chain subunits were biosynthesized in a relatively constant state (Fig. 1x). One of the two IPF drugs, pirfenidone (PFD), was able to prevent the progression of PF by efficiently rescuing complex V activity (Supplementary Fig. 11).

Here, we found that elevated TGF-β expression was associated with impairment of complex V activity in IPF and CTD-PF patients’ blood as well as in a BLM-induced PF mouse model. Further mechanistic study in human pulmonary epithelial cells revealed a cyto-/mito-translation imbalance. Specifically, we observed mTOR activation but cyto-translation damage and protein product instability, in contrast to mito-translation stability, that impaired the balanced biogenesis of multisubunit RCCs, mainly complex V, in the mitochondria of fibrotic cells. Decreases in complex V and ATP and a burst of ROS induced alveolar epithelial cell dedifferentiation.^5^ On the basis of these findings, TGF-β + /complex V− may be a potential biomarker of PF and a drug target for pulmonary regeneration in the post-COVID era.

## Supplementary information

Supplementary Materials

Video S1-3D-1

Video S1-3D-2

Video S1-3D-3

Video S1-3D-4

Video S1-3D-5

Video S1-Time-1

Video S1-Time-2

Video S1-Time-3

Video S1-Time-4

Video S1-Time-5

## Data Availability

The raw data from the RNA-Seq analysis have been deposited in the Sequence Read Archive (SRA) database (Accession number: PRJNA932932). The data supporting the findings of this study are available from the corresponding author upon reasonable request. Source data are provided with this paper.

## Code availability

All codes in this study are available upon reasonable request.
