# Supplementary material for: TGF-β changes cyto/mito-ribosome balance to target respiratory chain complex V biogenesis in pulmonary fibrosis therapy
Source: Signal Transduct Target Ther. 2023 Mar 21;8:136. doi: 10.1038/s41392-023-01370-2 (PMC10030894; doi:10.1038/s41392-023-01370-2)
Supplement: Supplementary file 1 — Supplementary Materials [file 41392_2023_1370_MOESM1_ESM.doc]

Supplementary Materials for

**TGF-β changes cyto/mito-ribosome balance to target respiratory chain complex V biogenesis in pulmonary fibrosis therapy**

Na Zhang^1,†^, Zunling Zhao^2,3, †^, Yin Zhao^1, †^, Lei Yang^2,3, †^, Yanhong Xue^2^, Yun Feng^2^, Jianjun Luo^2,3^, Runsheng Chen^2,3^, Wei Wei^1^ and Yan Qin^2,3^,*

**Author affiliations and contact information**

^1^Department of Rheumatology and Immunology, Tianjin Medical University General Hospital, Tianjin 300052, P. R. China

^2^CAS Center for Excellence in Biomacromolecules, Institute of Biophysics, Chinese Academy of Sciences, 15 Datun Road, Chaoyang District, Beijing 100101, China

^3^University of Chinese Academy of Sciences, Beijing 100049, China

^†^ Na Zhang, Zunling Zhao, Yin Zhao and Yang Lei contributed equally to this work.

* To whom correspondence should be addressed. E-mail: [qiny@ibp.ac.cn](mailto:qiny@ibp.ac.cn)

**This PDF file includes:**

Materials and Methods

Figures S1 to S11

Table S1 to S3

Video S1

**MATERIALS AND METHODS**

**Human samples**

Adult IPF patients (N=9), patients with connective tissue disease-associated PF (CTD-PF) (N=41), and age- and sex-matched healthy controls (HCs) (N=9) were enrolled from the Tianjin Medical University General Hospital. All patients fulfilled the diagnostic criteria. All patients and HCs underwent chest HRCT. The HRCT of the HCs revealed no signs of lung fibrosis, while the HRCT of the PF patients enrolled in this study revealed a UIP pattern, which is a representative pattern of IPF patients, as previously mentioned. Blood samples were collected from the enrolled patients and HCs and stored at -80°C for further analysis. The study was approved by the local ethics committee (approval number IRB2021-YX-003-01). All participants provided written informed consent prior to study inclusion.

**Clinical and paraclinical assessment**

The data obtained included demographic information (age and sex), main symptoms and laboratory indicators. HRCT and pulmonary function tests (PFTs) were used to evaluate ILD. The CT machines were Siemens Shuangyuan, Philips 256 row and GE64 row. The scan ranges from the apex of the lung to the bottom of the lung. The thickness of the scanning layer was 1.25mm. Pulmonary window (window level -700HU, window width 1600HU) and mediastinal window (window level 50HU, window width 350HU) were used. For chest HRCT assessment, two experienced thoracic radiologists independently reviewed all chest HRCT images without knowledge of the patients’ clinical data. Based on published methods ^1^, HRCT scans were scored at five levels for total disease extent, extent of reticulation and proportion of ground glass. The HRCT scores were recorded as percentage of lesion coverage to total lung extent. The clinical characteristics of the patient population are summarized in Supplementary Table 1.

**Measurement of mitochondrial respiratory chain complex activity**

The activities of mitochondrial complex I, complex II, complex III, complex IV, and complex V in human blood samples were determined by a Micro Mitochondrial Respiratory Chain Complex Activity Assay Kit (Solarbio, China) according to the manufacturer’s instructions. In brief, mitochondrial homogenates were added to the respective reaction buffer. The reaction mixture was transferred to a prewarmed (30 °C) quartz cuvette and immediately placed in a spectrophotometer. The absorbance of the reaction mixture was measured at 340 nm for complex I, 605 nm for complex II, 550 nm for complex III, 550 nm for complex IV, and 660 nm for complex V. Mitochondrial complex activity is expressed as U/mg prot.

**Determination of** **plasma PDGF-BB, IL-6, IL-8, TGF-β**

The plasma samples were available. Plasma PDGF-BB, IL-6, IL-8, TGF-β levels were quantified by ELISA according to the manufacturer’s instructions (Human PDGF-BB, TGF-β kit, IL-6, IL-8 from R&D Systems, Wiesbaden, Germany).

**Animal experiments**

All studies were performed according to the guidelines of the institutional biomedical research ethics committee, experimental animal center, Institute of Biophysics, Chinese Academy of Sciences (permission number: SYXK2021-121), China. All mice were housed in a pathogen-free facility. All applicable institutional and/or national guidelines for the care and use of animals were followed. All mice were obtained from the Department of Laboratory Animal Science, Peking University Health Science Center.

**Bleomycin-induced lung fibrosis mouse**

Thirty-two 8-week-old C57BL/6 mice were divided into two groups: a bleomycin-treated group and a control group. The mice were anesthetized intraperitoneally with 1.25% tribromoethanol (200 μl/10 g) and then administered a single dose of intratracheal bleomycin hydrochloride (Hanhui Pharmaceutical company, China) ^2-5^ at a concentration of 2.5 mg/kg (3.5 mg/ml in PBS) in the bleomycin-treated group ^6^ and the same amount of PBS in the control group. Mice were sacrificed by cervical dislocation at 14 days, and lung tissue was dissected and removed.

**H&E and Masson’s trichrome staining**

The lung tissues of mice were collected and fixed in 4% paraformaldehyde for one week, embedded in paraffin and stained with hematoxylin-eosin (H&E) and Masson’s trichrome. The lung sections were observed by a pathological section scanner (Leica CS2, Germany), and the degree of pulmonary fibrosis in each group was evaluated according to the imaging data. In addition, other tissues of mice, such as the heart, liver, spleen and kidney, were observed by H&E staining.

**Immunohistochemistry**

Automatic immunohistochemistry and an *in-situ* hybridization staining apparatus (Leica, Germany) were then conducted in paraformaldehyde-fixed, paraffin-embedded lung sections to detect the expression of proteins of interest. The antibodies used were TGF-beta 1 (Proteintech, 21898-1-AP, 1:200 dilution), collagen 1 (Proteintech, 67288-1-Ig, 1:200 dilution), PI3K (Proteintech, 20584-1-AP, 1:200 dilution), S6 (Cell Signaling, 2217S, 1:200 dilution), MRPL48 (Abcam, ab194826, 1:150 dilution), and ATP5a (Proteintech, 66037-1-Ig, 1:200 dilution).

**Isolation of Primary Type II Alveolar Epithelial Cells (AECII)**

Primary AEC II cell was obtained from Dr. Xue Li (Tianjin Key Laboratory of Lung Regenerative Medicine, Tianjin University Haihe Hospital). The mice were intraperitoneally anesthetized with 1% sodium pentobarbital (50 mg/kg) and euthanized by exsanguination. After the exposure of thoracic cavity and trachea, lungs were perfused with PBS 1 X through tracheal intubation. Then lungs were resected en bloc. Then lung single-cell suspensions were generated by elastase digestion and stained for FACS as described previously ^7^. In brief, mouse lungs were perfused and minced in a solution with elastase (4 U/mL; Worthington Biochemical Corporation, Lakewood, NJ), followed by incubation with DNase I (100 U/mL; Sigma-Aldrich, St. Louis, MO) for 15 min at 37 ℃. After adding of 10ml cold Hank’s balanced salt solution (HBSS) (Cellgro) and DMEM/F12 mediam (Gibco), the resulting cell suspension was filtered through a 70-µm cell strainer (Falcon; BD Biosciences, San Jose, CA) for flow cytometry. Red blood cell lysis buffer was added to remove erythrocytes. Cell pellets were resuspended in HBSS supplemented with 2% FBS, 10 mM HEPES, 0.1 mM EDTA, 100 IU/mL penicillin, and 100 mg/mL streptomycin (HBSS-plus). Flow cytometry was performed with primary antibodies against CD31-biotin, CD34-biotin, CD45-biotin, CD24-phycoerythrin (PE), anti-epithelial cellular adhesion molecule (EpCAM)-PE-Cy7, and Sca-1-allophycocyanin. The secondary antibody was against streptavidin. All antibodies were from eBioscience (San Diego, CA). Cells were stained for approximately 45 min in the dark on ice. After one wash with HBSS-plus, 7-amino-actinomycin D was added to label dead cells. Flow cytometry analysis was performed using single-color controls for compensation and established gating strategies based on isotype-negative controls on a FACSAria III sorter (BD Immunocytometry Systems, San Jose, CA). Mouse AECII cells, defined as CD31CD34CD45(Lin) EpCAM+CD24Sca-1, were sorted in HBSS-plus for further experiments.

**Cell culture and group treatment**

HLF cell line was obtained from Dr. Guangbiao Zhou (Chinese Academy of Medical Sciences). A549, H1299 and 16HBE cells were obtained from Dr. Taotao Wei (IBP, CAS). All cell lines were cultured in Dulbecco’s modified Eagle’s medium (DMEM; BI) supplemented with 10% fetal bovine serum (FBS, BI) and 1% penicillin–streptomycin (Gibco, USA) at 37°C in a 5% CO_2_ incubator. The cells were starved in serum-free DMEM supplemented with 1% penicillin–streptomycin for 24 h. Then, the cells were incubated with different treatments as follows.

Treatment 1: A549, H1299, 16HBE and HLF cells were treated with different concentrations of TGF-β1 (1, 2, 5, 10, 20, or 30 ng/ml), and ACEII were treated with TGF-β1 10 ng/ml, and the 0 ng/ml TGF-β1 group was used as the control group and cultured for 48 h.

Treatment 2: A549 cells were treated with different concentrations of TGF-β1 (0 or 10 ng/ml), and the 0 ng/ml TGF-β1 group was used as the control group and cultured for 24, 48 or 72 h.

Treatment 3: A549 cells were treated with 5 ng/ml TGF-β1 (T group), 5 ng/ml TGF-β1 + 250 μg/ml PFD (T-P1 group), 5 ng/ml TGF-β1 + 500 μg/ml PFD (T-P2 group) and 500 μg/ml PFD (P2 group). Normal cultured cells were used as the control group and cultured for 48 h. The PFD API was obtained from Beijing Continent Pharmaceuticals Co.,Ltd.

**Cellular morphology and mitochondrial morphology observation**

*Cellular morphology.* Cells cultured under the two treatment conditions were photographed under 10× bright field microscopy by inverted fluorescence microscopy (mshot, China) to observe the changes in cell morphology.

*Structure illumination microscopy (SIM).* Cells were cultured in a glass-bottom confocal culture dish. After the treatment, the cells were maintained in fresh medium. Then, 100 nM MITO tracker Green (Invitrogen, USA) was added to stain the cells at 37°C for 20 min in the dark. After washing with PBS, fresh medium and two drops per ml of NucBlue™ Live ReadyProbes™ reagent (Invitrogen, USA) were added for 10 min in the dark at 37°C. Then, the cells were washed three times with PBS and maintained in fresh medium for further imaging. Structure illumination microscopy (SIM, Applied Precision) was used to capture the 4D mitochondrial dynamics of 2 μm cells separated by 2 S for 1 min in OMX mode.

*Transmission electron microscopy (TEM).* Cells with different treatments were washed with PBS and then collected in centrifuge tubes. Then, the cells were fixed with 2.5% glutaraldehyde and postfixed with 3% osmium tetroxide. The specimens were dehydrated in a graded series of ethanol, embedded in Epon resin, sectioned and then imaged under a transmission electron microscope.

**Mitochondria extraction**

For mitochondrial extraction, cells exposed to different stimuli were washed with precooled PBS three times, scraped in PBS and collected by centrifugation. Then, the pellets were resuspended in 200 μl of precooled PBS and an equal volume of 4 mg/ml digitonin (dissolved in PBS) and incubated on ice for 10 min. After washing twice with PBS to remove residual digitonin, the pellets were resuspended in 50 μl of BN sample buffer and 5 μl of 10% DDM and incubated on ice for 20 min. Finally, after centrifugation at 20,000 g at 4°C for 20 min, 5 μl of the supernatant was retained for BCA protein quantification, and the remainder was mixed with 5% Coomassie Brilliant Blue G-250 and stored at -80°C for subsequent use.

**BN-PAGE and gel activity**

Mitochondria extraction samples were loaded onto blue-native gradient gels (6-15% nondenaturing gradient gels). Then, BN-PAGE was performed with a constant current of 8 mA. The detection of mitochondrial complexes was carried out after BN-PAGE by staining gels with Coomassie Brilliant Blue and observation after decolorization. Then, in-gel activity staining of the complexes was assessed using dyes and substrates specific for each complex. After color development, the gels were washed with distilled water, and then, the reactions were stopped by adding 2-4% glacial acetic acid. Usually, this process requires 15 min-30 min for complexes I, II and IV and several hours for complex V.

**Lung tissue protein extraction**

The lung tissue was removed from the mouse body and washed with PBS 3 times. After taking pictures, scissors were used to cut the tissue approximately 3 mm in diameter. After PBS cleaning, the tissue was placed into a 1.5 ml centrifuge tube, and 500 μl RIPA buffer (including protease and phosphatase inhibitors) was added and placed on ice. The tissue was minced with scissors, and 30 min was placed on ice and then centrifuged at 14000 rpm and 4 ℃ for 15 min. The supernatant was placed in a new 1.5 ml centrifuge tube, and the protein concentration was determined by the bicinchoninic acid (BCA) method. The samples were stored at -20 ℃.

**Western blot (WB) analysis**

Mitochondria extraction was added with 5% β-mercapto-ethanol to the sample buffer and boiled at 98°C for 5 min prior to sample loading. The polyacrylamide gel was prepared using a one-step 10% SDS–PAGE gel rapid preparation kit (Biotides, China). Electrophoresis was performed at 120 V for 90 min. The gels were transferred to a polyvinylidene difluoride (PVDF) membrane using the wet transfer method, and electrophoresis was performed at 350 mA for 90 min. The membranes were blocked, incubated with primary antibodies against NDUFA9 (Abcam, ab128744, 1:1000 dilution), SDHA (Abcam, ab14715, 1:1000 dilution), UQCRC2 (Abcam, ab203832, 1:1000 dilution), COX4 (Abcam, ab110272, 1:1000 dilution), ATP5A (Abcam, ab176569, 1:1000 dilution), ATP6 (Beyotime, AF6261, 1:1000 dilution), ATP6V0D1 (Abcam, ab202897, 1:1000 dilution), ATP8 (Proteintech, 26723-1-AP, 1:1000 dilution), phospho (Proteintech, 60004-1-IG, 1:1000 dilution), AKT (Cell Signaling, 4691T, 1:1000 dilution), phosphor-AKT (Ser473) (Cell Signaling, 4060T, 1:1000 dilution), mTOR (Cell Signaling, 2983S, 1:1000 dilution), PI3K (Cell Signaling, 4292S, 1:1000 dilution), phospho-P70S6K (Thr389) (Cell Signaling, 9205S, 1:1000 dilution), phospho-S6 (Ser235/236) (Cell Signaling, 4858T, 1:1000 dilution), S6 (Cell Signaling, 2217S, 1:1000 dilution), phospho-EIF2S1 (Ser51) (Cell Signaling, 3398T, 1:1000 dilution), MRPL48 (Abcam, ab194826, 1:1000 dilution), MRPL28 (Abcam, ab126719, 1:1000 dilution), MRPL18 (Abcam, ab67844, 1:1000 dilution), MRPS35 (Abcam, ab175931, 1:1000 dilution), MRPS18B (Abcam, ab191891, 1:1000 dilution), TOM20 (Abcam, ab186735, 1:1000 dilution) and GAPDH (Proteintech, 60004-1-IG, 1:1000 dilution) for 1 h at 37℃, washed with TBST, and incubated with a secondary antibody (Beyotime, China) for 1 h at 37℃. After washing the membranes with TBST, the membranes were analyzed using a ChemiDoc™ MP system.

**ATP detection**

ATP was extracted according to the operation instructions of a commercial ATP detection kit (Beyotime, China), and a multifunction microplate reader was used for detection with a luminometer.

**Cellular ROS and mitochondrial ROS**

Cells pretreated as mentioned above were digested with 0.05% trypsin, collected in a 1.5 ml centrifuge tube, washed once with PBS, and processed according to the operating instructions of a Reactive Oxygen Species Assay Kit (Beyotime, China) and MitoSOX™ Red Mitochondrial Superoxide Indicator (Invitrogen). Cellular ROS and mitochondrial ROS were analyzed by flow cytometry (BD, USA).

**TMRE**

The mitochondrial membrane potential was evaluated using TMRE (Sigma, 87917). The cells were cultured in confocal dishes as before. The TMRE was stored in a 5 mM solution prepared with DMSO (BBI, A600163). To prepare the TMRE working solution, the TMRE storage solution was diluted with PBS to 500 nM. The cells were stained with the TMRE working solution for 10 min at 37°C and protected from light exposure. After washing with PBS three times, fresh medium was added. The images were taken using an OLYMPUS FV3000 with a 60x oil-immersion objective under the same parameter settings.

**RNA-seq analysis**

Cells were washed with PBS after trypsin digestion and centrifuged at 1,200 g at 4°C for 3 min to obtain the cell precipitate, and then, total RNA was extracted according to the TRIzol kit instructions (Invitrogen, USA). A nanophotometer spectrophotometer (IMPLEN, CA, USA) and Agilent 2100 Bioanalyzer (Agilent Technologies, CA, USA) were used to detect the purity and integrity of the RNA. The cDNA library was established using a NEBNext® UltraTM RNA Library Prep Kit for Illumina® (NEB, USA). After the cDNA Library was built, a Qubit 2.0 Fluorometer was used for the initial quantification. The library was diluted to 1.5 ng/µl, and then, an Agilent 2100 Bioanalyzer was used to test the insert size of the library. After the insert size met the expectation, qRT–PCR was used to accurately quantify the library effective concentration (library effective concentration was higher than 2 nM) to ensure library quality. Illumina sequencing was performed after passing the library inspection. To ensure the quality and reliability of the data analysis, the original data were filtered. Filtering mainly included the removal of reads with adapters, reads containing N (N represents uncertain base information) and low-quality reads (Qphred ≤20 represents more than 50% of the reads of the entire read length). Furthermore, the Q20, Q30 and GC contents were calculated based on the clean data. All subsequent analyses were high-quality analyses based on clean data.

The clean reads were mapped to the human genome version GRCh38 using STAR. Genes were identified using a GTF obtained from Ensembl release-104. Each gene read was counted using RSEM. The normalization of the read counts and p values of the differentially expressed genes were computed using DEseq2. GO enrichment and KEGG pathway analyses of genes were performed by using the R package clusterProfiler. Volcano plots displaying the expression changes in the genes were generated using the R package ggplot2.

**Quantitative PCR**

We first extracted the total RNA from cells with a TRIzol kit (Invitrogen, USA), reverse transcribed the cDNA with a PrimeScript™ RT reagent Kit (TAKARA, Japan), and performed qPCR verification according to the instructions for TB Green Premix Ex Taq II (Tli RNaseH Plus) (TAKARA, Japan).

**Polysome**

Two buffers were prepared. The first buffer was 10× lysis buffer prepared as follows: 50 mM Tris-HCl (pH 7.5), 25 mM MgCl_2_, and 15 mM KCl. The second buffer was polysome buffer prepared as follows: 100 μl of 10× lysis buffer, 10 μl of 10 mg/ml CHX (100 μg/ml), 1 μl of 1 M DTT (1 mM), 10 μl 100× cocktail (EDTA-free, Roche), 50 μl of 10% Triton X-100, 50 μl of 10% sodium deoxycholate, RNase inhibitor, and up to 1 ml of DEPC water.

Cells were transferred to a 10 cm^2^ petri dish, and then 100 µg/ml CHX was added. After incubation for 10 min, the cells were washed with cold PBS three times. The cells were scraped in PBS and collected by centrifugation. Then, the pellets were resuspended in 1 ml polysome buffer and incubated on ice for 15 min. Then, the supernatant was collected by centrifugation at 13000 rpm for 20 min at 4°C. Loading was determined based on the A260 absorbance by a Nanodrop spectrophotometer. The sample was gently added to the upper surface of a prechilled 0-50% sucrose gradient (1× lysis buffer) in an SW40 centrifuge tube and then centrifuged with ultracentrifugation at 36,000 rpm and 4°C for 3 h; finally, a nucleic acid protein detector was used to detect the sample.

**Immunofluorescence**

The cells were cultured in confocal dishes as previously described, the cell culture medium was removed, and the cells were rinsed three times with 500 µL of PBS. Then, we used 400 µL of 4% paraformaldehyde (Beyotime, China) for 15 min at 37°C to fix the cells. The paraformaldehyde solution was removed, and the cells were washed three times with 1 ml of PBS. After adding 1 ml of 0.1% Triton X-100 in PBS and incubating the cells at room temperature for 20 min, Triton X-100 was removed, and the cells were washed three times with 1 ml of PBS. Then, 500 µL of QuickBlock™ Blocking Buffer for Immunol Staining (Beyotime, China) was added, and the cells were incubated at room temperature for 60 min. The blocking buffer was removed, and primary antibodies against ATP5A (Abcam, ab176569, 1:1000 dilution), ATP6 (Beyotime, AF6261, 1:1000 dilution), MPRL48 (Abcam, ab194826, 1:1000 dilution), MRPS35 (Abcam, ab175931, 1:1000 dilution), RPS6 (Abcam, ab225676, 1:1000 dilution), and rRNA (Santa Cruz Biotechnology, sc-33678) diluted in 500 µL of QuickBlock™ Primary Antibody Dilution Buffer for Immunol Staining (Beyotime, China) were added to the cells and incubated overnight at 4°C. The primary antibody solution was removed, and the cells were washed three times with 1 ml of PBS. The desired concentration of a fluorescent dye–labeled secondary antibody was added to 500 µL of QuickBlock™ Secondary Antibody Dilution Buffer for Immunofluorescence (Beyotime, China), and the cells were incubated for 1 h at room temperature protected from light. The solution was removed, and the cells were rinsed three times with 1 ml of PBS. Next, 200 µL of DAPI was added, and the cells were incubated for 10 min at room temperature protected from light. The cells were again rinsed three times with 1 ml of PBST. Finally, we added an appropriate amount of Antifade Mounting Medium (Beyotime, China). Images were taken using an OLYMPUS FV3000 with a 60x oil-immersion objective under the same parameter settings.

**Protein synthesis labeling by click-iT l-azidohomoalanine (AHA)**

The cell treatment was the same as described above. The medium was first removed, and the cells were incubated with L-Met minus medium at 37°C at 5% CO_2_ for 30 min, which was replaced with L-Met minus and L-AHA (50 µM) plus medium at 37°C at 5% CO_2_ for 1 h. Then, one group directly progressed to the next step, while in the other groups, fresh L-Met medium without L-AHA was added, followed by culture for 1 h and 2 h before proceeding to the next step. The cell pellet was collected and washed with cold PBS and lysis buffer (50 mM Tris·Cl pH 8.0, 1% SDS). The cell lysate was transferred to a 1.5 ml tube and sonicated for 5 min. Then, 50 μl protein samples were collected, and biotin-alkyne was added to a final concentration of 100 µM. The samples were vortexed, TCEP was added to a final concentration of 1 mM, and THPTA was added to a final concentration of 100 µM. The samples were vortexed again, and CuSO_4_ was added to a final concentration of 1 mM, followed by incubation for 1.5 h at 25°C. Then, 600 µl of methanol was added, the samples were vortexed, 150 µl of chloroform was added, the samples were vortexed, and 400 µl of H_2_O was added. The samples were centrifuged at 18,000 × g for 5 min at 4°C. The upper liquid layer was removed, and 500 µl of methanol was added. The samples were again centrifuged at 18,000 × g for 5 min at 4°C. The upper liquid layer was removed, and the samples were air dried for approximately 15 min. Finally, SDS loading buffer was added for WB analysis.

**OP-puromycin (OPP) staining**

Cells were treated with either PBS or 30 μM OPP (APExBIO, A8778-5) for 5 min with intermittent gentle agitation. Then, the cells were washed with PBS and fixed with cold methanol for 2 min at 20°C. The cells were washed with Tris-buffered saline (TBS), permeabilized with TBST (TBS with 0.2% Triton X-100) for 20 min at room temperature, and then washed with TBS again. Then, the cells were incubated with Alexa 448-azide (1:1000), 1 mM TCEP, 100 μM TBTA, and 1 mM CuSO4 for 1 h without light exposure and washed with TBS.

**Pico green staining to detect mitochondrial DNA**

The cells were cultured in confocal dishes and washed with PBS three times. Then, the cells were incubated with Pico green (diluted with medium at a ratio of 1:500, Invitrogen, P11496) for 30 min at 37°C. After washing with PBS three times, the cells were stained with 100 nM MITO tracker Red (Invitrogen, USA) in fresh medium for 20 min in the dark at 37°C. Then, the cells were washed with PBS and maintained in fresh medium, and two drops of NucBlue™ Live ReadyProbes™ reagent (Invitrogen, USA) per ml were added to stain the cells for 10 min in the dark at 37°C. After washing with PBS three times and adding fresh medium, images were taken using an OLYMPUS FV3000 with a 60x oil-immersion objective under the same parameter settings.

**Statistical analysis**

Regarding the description of the quantitative data, the normally distributed groups are described as the mean ± standard deviation, and the skewed distribution groups are described by the median [25th percentile, 75th percentile], whereas the categorical variables are presented as the number positive/number tested. The group comparisons were performed using Student’s *t test*, Wilcoxon rank sum, χ^2^ statistics, Fisher’s exact test and one-way ANOVA as appropriate. The correlations were tested using the Spearman test. To analyze the diagnostic value of respiratory chain complex activity for ILD, a receiver operating characteristic (ROC) curve analysis was conducted. A *p value* less than 0.05 was considered statistically significant. All statistical analyses were performed using SPSS version 22.0 (IBM Japan, Tokyo, Japan) and GraphPad Prism software 8.0 for Windows (GraphPad Software). All graphs were generated using GraphPad Prism software 8.0 for Windows (GraphPad Software, San Diego, California, USA).

**REFERENCES**

1 Goh, N. S. *et al.* Interstitial lung disease in systemic sclerosis: a simple staging system. *Am J Respir Crit Care Med.*  **177**, 1248-1254 (2008).

2 De langhe, E. *et al.* Quantification of Lung Fibrosis and Emphysema in Mice Using Automated Micro-Computed Tomography. *Plos One.* **7**, e43123 (2012)*.*

3 Ask, K. *et al.* Comparison between conventional and "clinical" assessment of experimental lung fibrosis. *J Transl Med.* **6**, 16-26 (2008).

4 Degryse, A. L. and Lawson, W. E. Progress Toward Improving Animal Models for Idiopathic Pulmonary Fibrosis. *Am J Med Sci.* **341**,444-449 (2011).

5 Ruscitti, F. *et al.* A Multimodal Imaging Approach Based on Micro-CT and Fluorescence Molecular Tomography for Longitudinal Assessment of Bleomycin-Induced Lung Fibrosis in Mice. *J Vis Exp.* **134**, 56443. (2018).

6 Mecozzi, L. *et al.* In-vivo lung fibrosis staging in a bleomycin-mouse model: a new micro-CT guided densitometric approach. *Sci Rep.* **10** (1), 18735. (2020).

7 Chen, H. *et al.* Airway epithelial progenitors are region specific and show differential responses to bleomycin-induced lung injury. *Stem Cells.* **30**, 1948-1960 (2012).

**Supplementary figures**


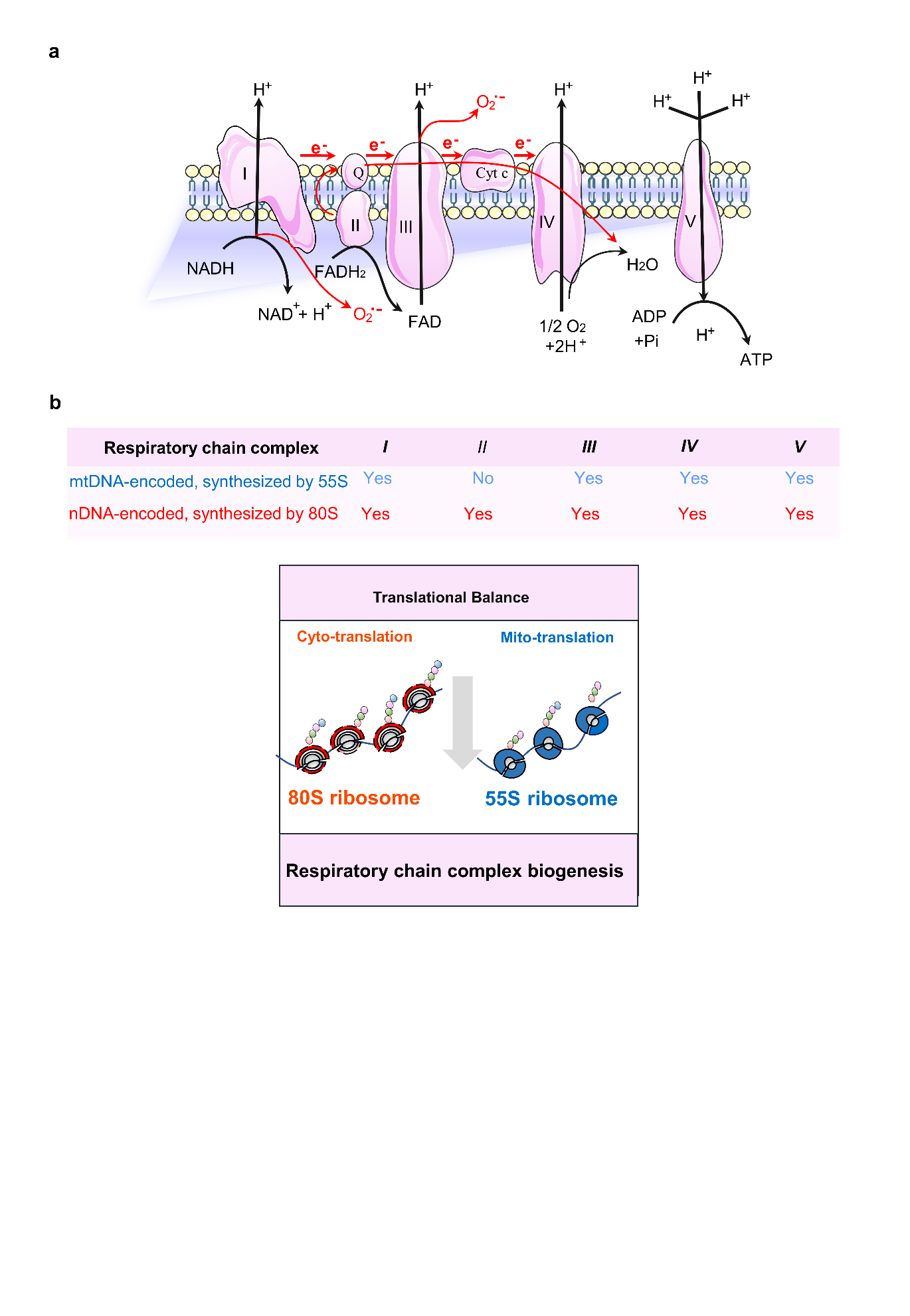


**Figure S1. Respiratory chain complex and biogenesis by co-ordination between the two translation systems**. **(a)** Respiratory chain complexes, located on the mitochondrial inner membrane, catalyze the electron transfer pathway between oxidation of NADH and succinate and the reduction of oxygen to water. Complex V couples the proton flow with the conversion of ADP and inorganic phosphate to ATP by using the proton electrochemical gradient across the inner mitochondrial membrane generated by respiratory chain complexes I – IV. **(b)** Respiratory chain complexes I, III, IV and V comprise both nuclear DNA (nDNA)- and mitochondrial DNA (mtDNA)-encoded subunits, and their biogenesis depends on the cooperation of cyto- and mito-translation.


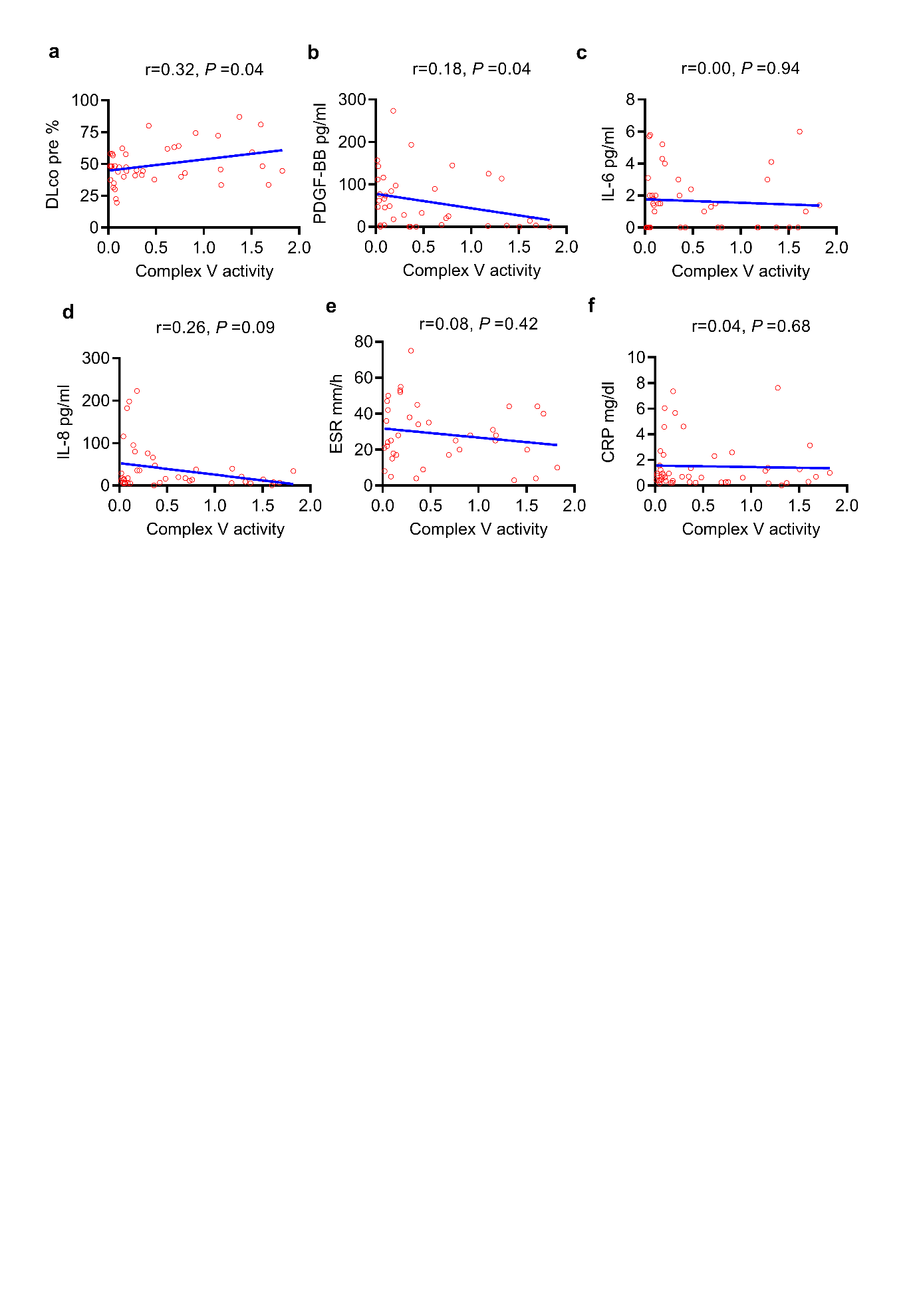


**Figure S2. Complex V activity levels don’t correlate with the inflammatory reaction of patients**. Correlation analysis between complex V activity and **(a)** DLco pre%, **(b)** PDGF-BB, **(c)** IL-6, **(d)** IL-8, **(e)** ESR, **(f)** CRP (*n* = 50).


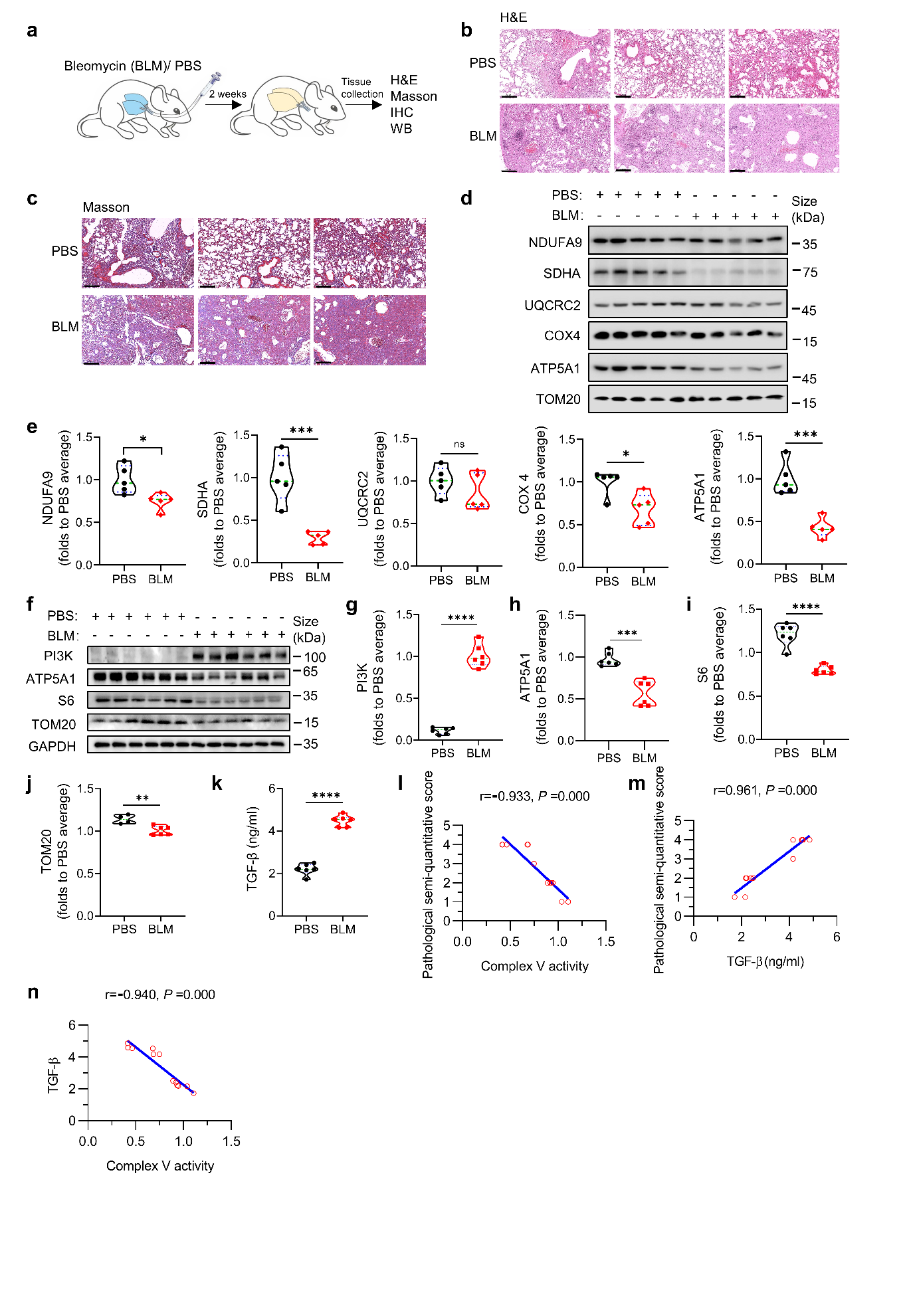


**Figure S3. Decreased expression of mitochondrial respiratory chain complex in mouse model of pulmonary fibrosis. (a)** Schematic diagram of constructing a mouse model of pulmonary fibrosis. Hematoxylin and eosin (H&E) and Masson staining, immunohistochemical (IHC), western blotting (WB) assays were performed. **(b)** Histopathological images of lung sections of the mice model. Scale bar = 200 μm (*n* = 5). **(c)** Histopathological images of masson-stained lung sections of the mice model. Scale bar = 200 μm (*n* = 5). **(d-e)** Immunoblot for protein expression levels of NDUFA9, SDHA, UQCRC2, COX4, ATP5a and TOM20 in lung tissue of mouse model **(d)** and quantification of each protein **(e)** (*n* = 5). **(f-j)** Immunoblot for protein expression levels of PI3K, ATP5A1, S6, TOM20 and GAPDH in blood of mouse model **(f)** and quantification of each protein **(g-j)** (*n* = 6). All lanes were loaded with the same amount of total protein and quantifications were made by the grayscale value of WB strips measured by Image J. Significance was determined using *t* test. **(k)** Concentrations of TGF-β in the blood of mice were detected using ELISA. (l) Correlation analysis between complex V activity and pathology semiquantitative score. (m) Correlation analysis between TGF-β and pathology semiquantitative score. (n) Correlation analysis between complex V activity and TGF-β. **P*<0.05; ***P* < 0.01, ****P*<0.001, *****P*<0.0001.


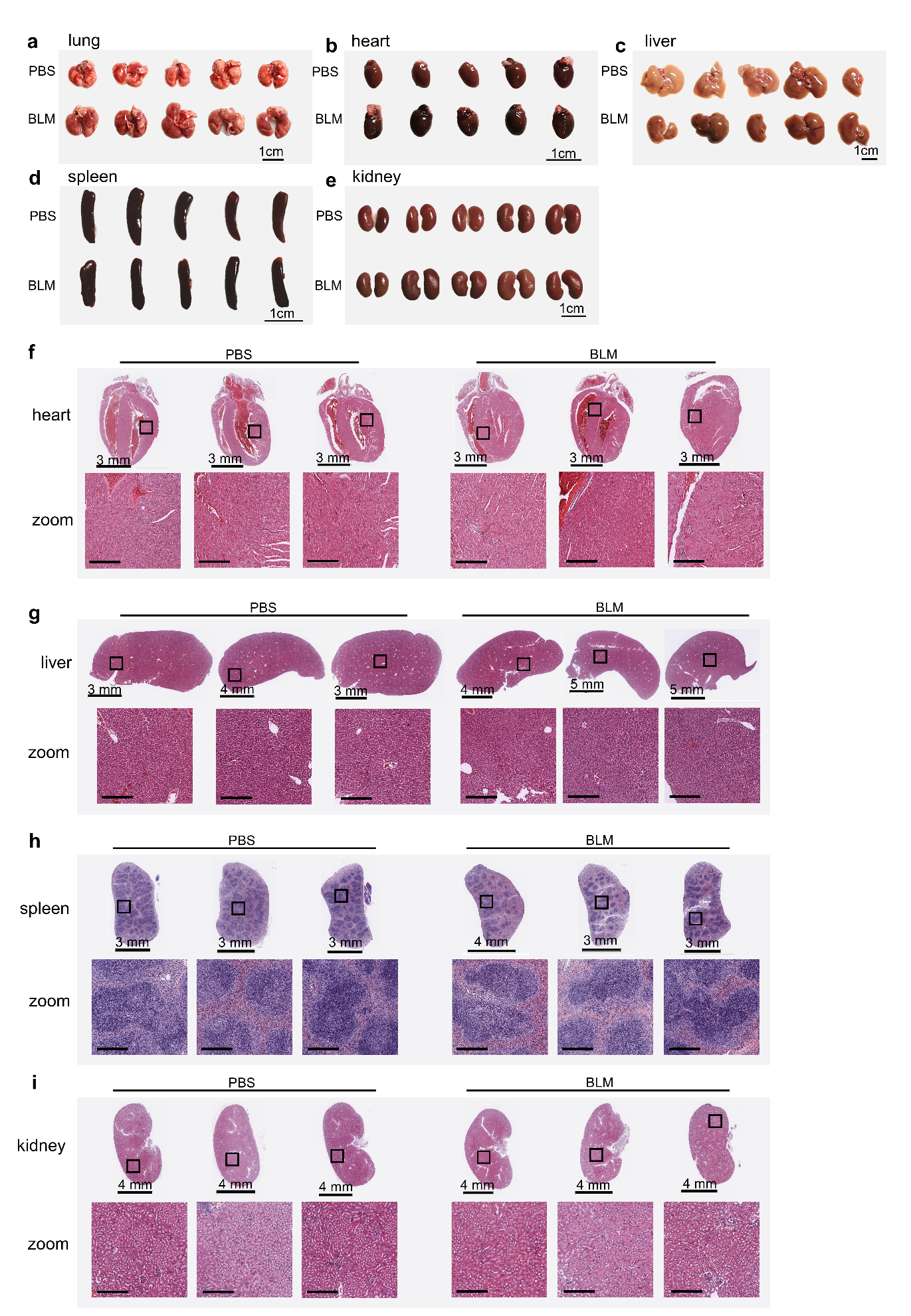


**Figure S4. Pathological analysis of main organs in lung fibrosis mice. (a-e)** Photograph of the lung, heart，liver，spleen and kidney in the phosphate-buffered saline (PBS) group and the bleomycin (BLM) treatment group (*n* = 5). **(f-l)** Histopathological images of hematoxylin and eosin-stained heart，liver，spleen and kidney sections of the mice model, Zoom: scale bar = 200 µm (*n* = 5).


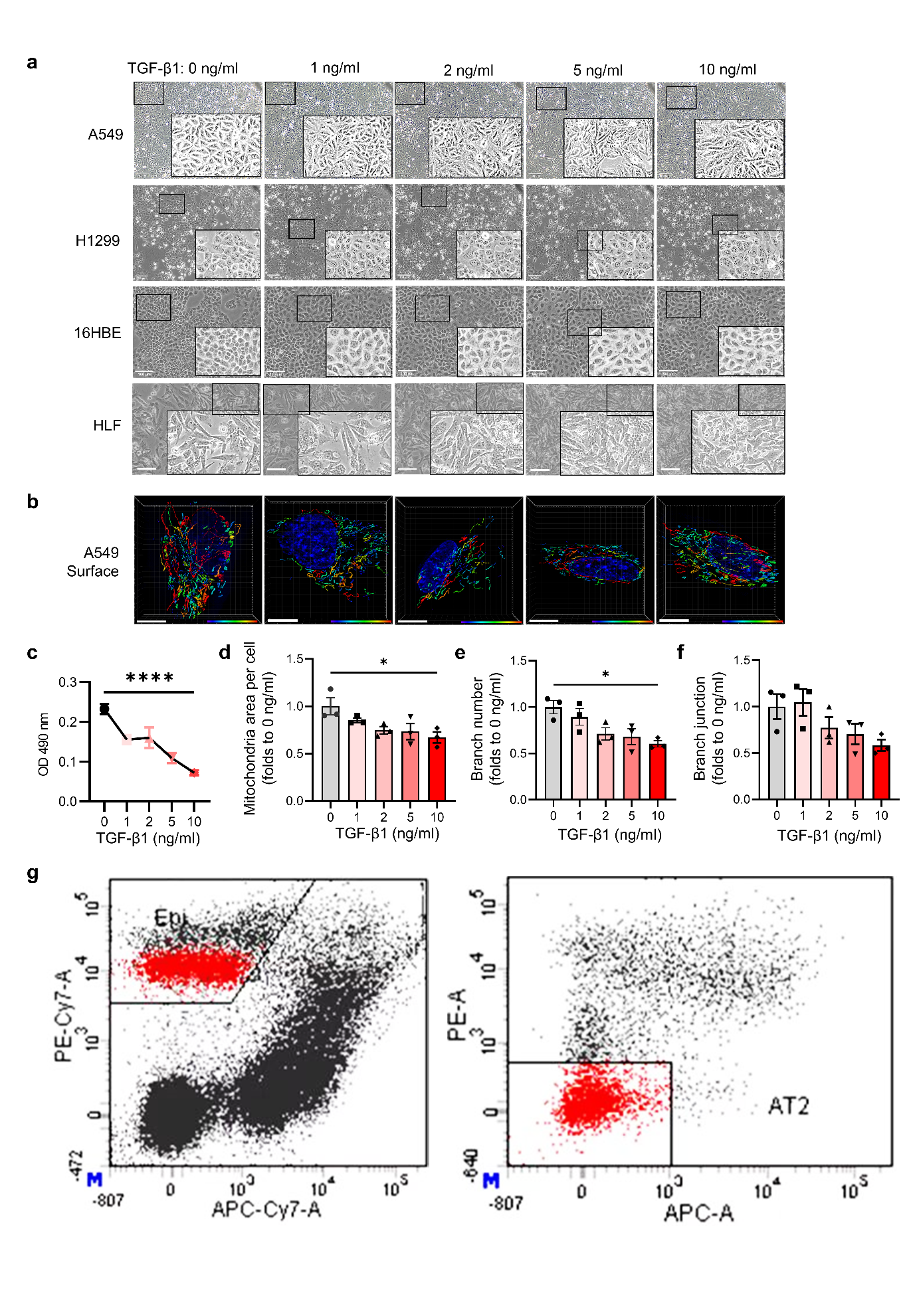


**Figure S5. Cellular and mitochondrial morphological changes of pulmonary epithelial cells induced by TGF-β. (a)** A549 cells, H1299, 16HBE and HLF cells were treated with different concentrations of TGF-β1 for 48 hours, and images were taken using Inverted fluorescence microscope. DIC images show the morphologic changes of cells under different treatment conditions and the images were taken using Nikon Ts2 inverted microscope with a 10x objective, scale bar = 100 μm (*n* = 3). **(b)** A549 cells were treated with different concentrations of TGF-β1 for 48 hours, and images were taken using OMX mode of structural light illumination microscope. The 3D images were processed by the OMX images using the Imaris software to build the surface (scale bar = 10 µm, color bar = Area, 0 – 20 μm^2^), and the parameters were the default values of the software (*n* = 3). **(c)** Proliferation A549 cells treated with different concentrations of TGF-β1 (*n* = 3). (**d-f)** Mitochondria Analyzer plug-in (default parameter) in Image J software was used to analyze the mitochondria area per cell, branches and branch junctions of Mitochondria in the 3D images and conduct data statistics (*n* = 3). **(g)** Mouse AECII cells, defined as CD31-CD34-CD45-(Lin-) EpCAM+CD24-Sca-1-, were sorted in HBSS-plus for further experiments. One-way ANOVA with Tukey’s multiple comparisons test was performed. **P*<0.05.


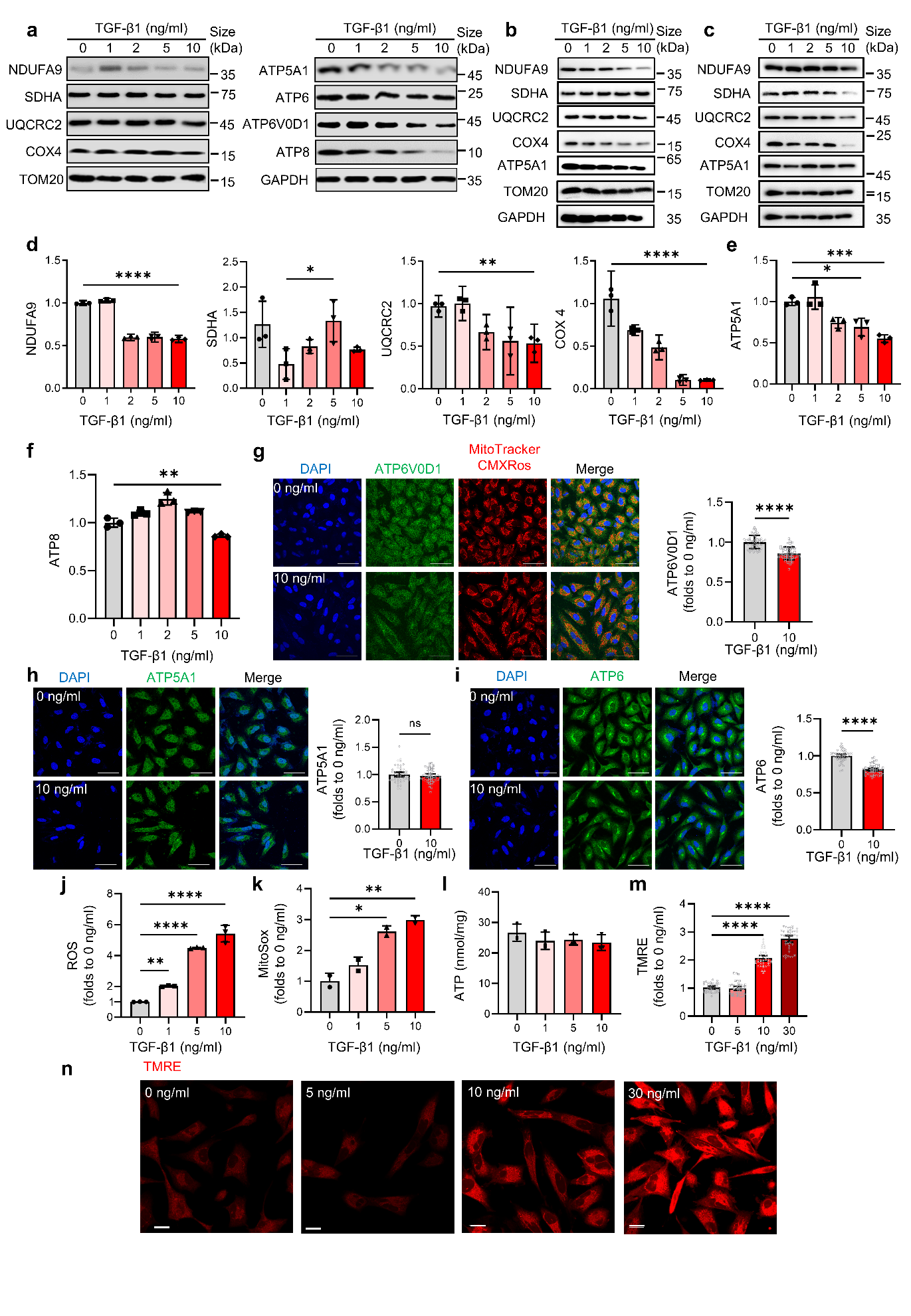


**Figure S6. Suppressed respiratory chain complex activity in TGF-β1 induced lung fibrosis.** **(a)** Immunoblot for protein expression levels of NDUFA9, SDHA, UQCRC2, COX4, ATP5a, ATP6, ATP6V0D1, ATP8, TOM20 and GAPDH in A549 lysates with different treatment conditions. All lanes were loaded with the same amount of total protein. **(b, c)** Immunoblot for protein expression levels of NDUFA9, SDHA, UQCRC2, COX4, ATP5a, TOM20 and GAPDH in H1299 and 16HBE lysates with different treatment conditions. All lanes were loaded with the same amount of total protein. **(d-f)** The expression of NDUFA9, SDHA, UQCRC2, COX4 **(d)**, ATP5a **(e)**, ATP8 **(f)** in cells was detected by qPCR (*n* = 3). **(g-i)** The expression of ATP6V0D1, ATP5a and ATP6 in cells was detected by immunofluorescence. The images were taken using OLYMPUS FV3000 with a 60x oil-immersion objective under the same parameter settings, scale bar = 50 µm. The fold changes of ATP5a, ATP6 and ATP6V0D1 expression in A549 cells, the mean fluorescence intensity was obtained by Image J for statistics (*n* = 50). **(j, k)** Fold changes of ROS and Mitosox in A549 cells induced by TGF-β1. The mean fluorescence intensity of DCF and Mitosox in A549 cells via flow cytometry (*n* = 3). **(l)** ATP content in A549 cells with different treatment conditions (*n* = 3). **(m)** Fold changes of TMRE in A549 cells induced by TGF-β1, the mean fluorescence intensity was obtained by Image J for statistics (*n* = 40). **(n)** Representative living cell image of mitochondrial membrane potential was detected by TMRE in A549 cells treated with different TGF-β1 concentrations. The images were taken using OLYMPUS FV3000 with a 60x oil-immersion objective under the same parameter settings, scale bar = 50 µm. One-way ANOVA with Tukey’s multiple comparisons test was performed. **P*<0.05; ***P*<0.01; ****P*<0.001; *****P*<0.0001.


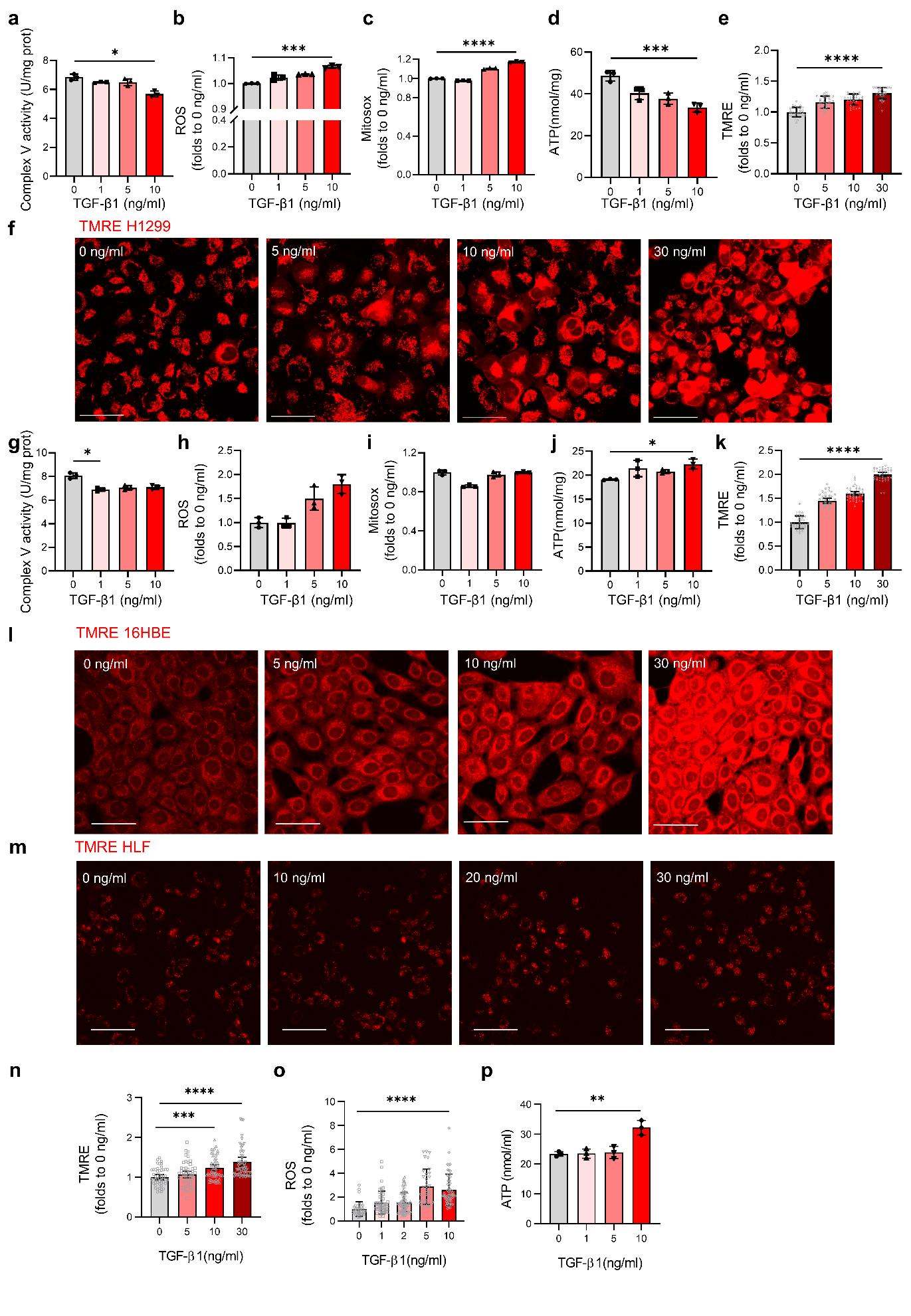


**Figure S7. TGF-β1 induces lung fibrosis through changed mitochondrial respiratory complex activity in H1299 and 16HBE cells. (a)** The activity of mitochondrial respiratory complex Ⅴ in H1299 cells with different treatment conditions (*n* = 3). **(b, c)** Fold changes of ROS and Mitosox in H1299 cells induced by TGF-β1. The mean fluorescence intensity of DCF and Mitosox in H1299 cells via flow cytometry (*n* = 3). **(d)** ATP content in H1299 cells with different treatment conditions (*n* = 3). **(e)** Fold changes of TMRE in H1299 cells, the mean fluorescence intensity was obtained by Image J for statistics (*n* = 30). **(f)** Representative living cell image of mitochondrial membrane potential was detected by TMRE in H1299 cells treated with different TGF-β1 concentrations. The images were taken using OLYMPUS FV3000 with a 60x oil-immersion objective under the same parameter settings, scale bar = 50 µm. **(g)** The activity of mitochondrial respiratory complex Ⅴ in 16HBE cells with different treatment conditions (*n* = 3). **(h, i)** Fold changes of ROS and Mitosox in 16HBE cells induced by TGF-β1. The mean fluorescence intensity of DCF and Mitosox in 16HBE cells via flow cytometry (*n* = 3). **(j)** ATP content in 16HBE cells with different treatment conditions (*n* = 3). **(k)** Fold changes of TMRE in 16HBE cells, the mean fluorescence intensity was obtained by Image J for statistics (*n* = 40). **(l, m)** Representative living cell image of mitochondrial membrane potential was detected by TMRE in 16HBE and HLF cells treated with different TGF-β1 concentrations. The images were taken using OLYMPUS FV3000 with a 60x oil-immersion objective under the same parameter settings, scale bar = 50 µm. **(n)** Fold changes of TMRE in HLF cells, the mean fluorescence intensity was obtained by Image J for statistics (n = 50). **(o)** Fold changes of ROS in HLF cells, the mean fluorescence intensity was obtained by Image J for statistics (n = 30). The images were taken using OLYMPUS FV3000 with a 60x oil-immersion objective under the same parameter settings. **(p)** ATP content in HLF cells with different treatment conditions (n = 3). One-way ANOVA with Tukey’s multiple comparisons test was performed. **P*<0.05; ****P*<0.001; *****P*<0.0001.


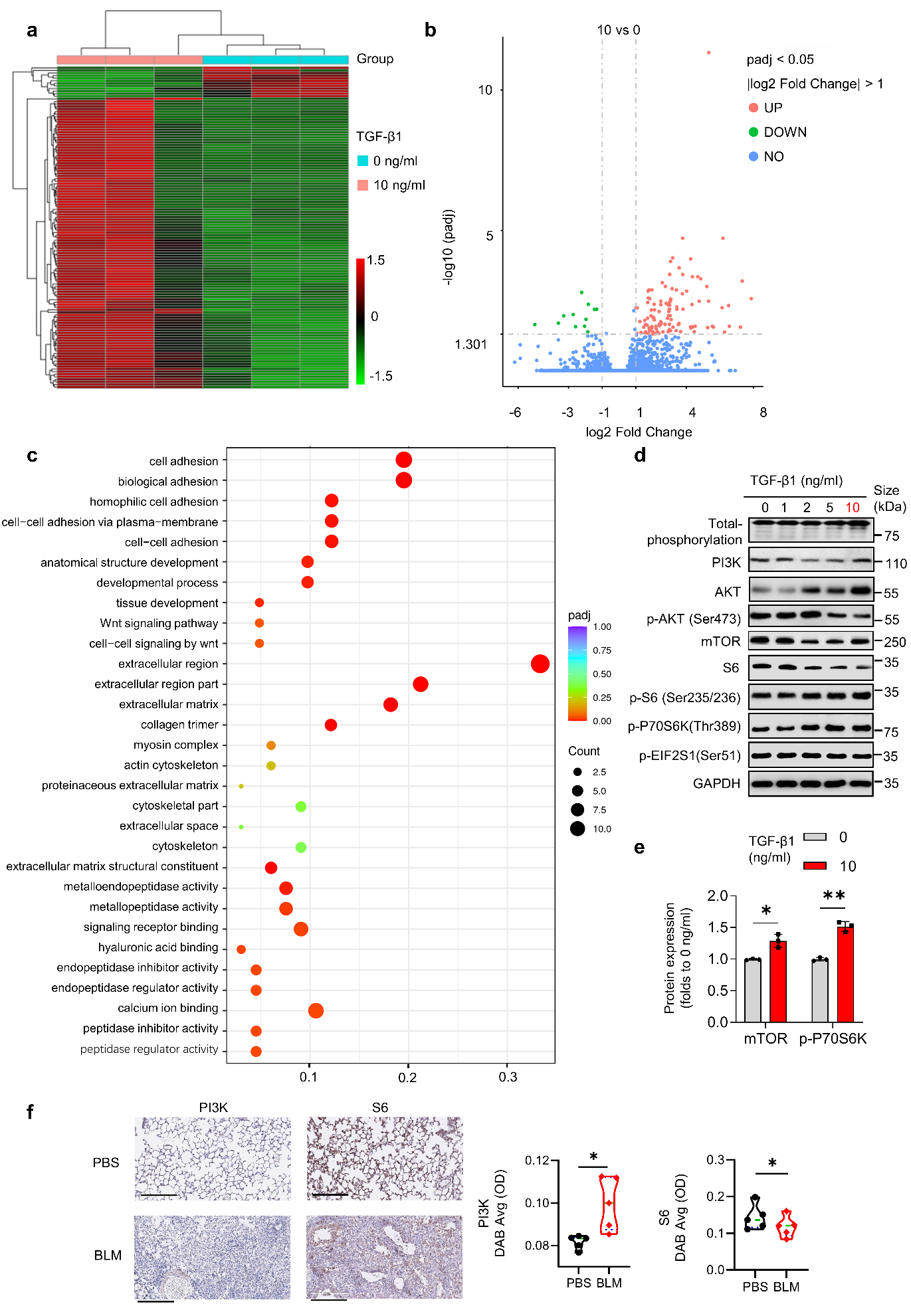


**Figure S8. PI3K-mTOR was the most upregulated pathway to activate cytoplasmic translation upon TGF-β-induced fibrosis.** **(a)** Differential expression of genes based on RNA-seq data in A549 cells induced by TGF 0 and 10 ng/ml (*n* = 3). **(b)** Volcano map analysis of differential gene expression (*n* = 3). **(c)** Annotations of transcripts in the GO library (*n* = 3). **(d)** Immunoblot for protein expression levels of Total- phosphorylation, AKT, phospho-AKT (Ser473), mTOR, PI3K, S6, phospho-S6 (Ser235/236), phospho-P70S6K (Thr389), phospho-EIF2S1 (S51) and GAPDH in A549 lysates with different treatment conditions. All lanes were loaded with the same amount of total protein. **(e)** Fold changes of mTOR and S6 protein expression, statistics were made by the grayscale value of WB strips measured by Image J (*n* = 3). **(f)** Immunohistochemical analysis of PI3K and S6 in lung tissue of mouse model (*n* = 5). Significance was determined using t test. ns: not significant; **P* < 0.05; ***P* < 0.01; ****P*<0.001; *****P*<0.0001.


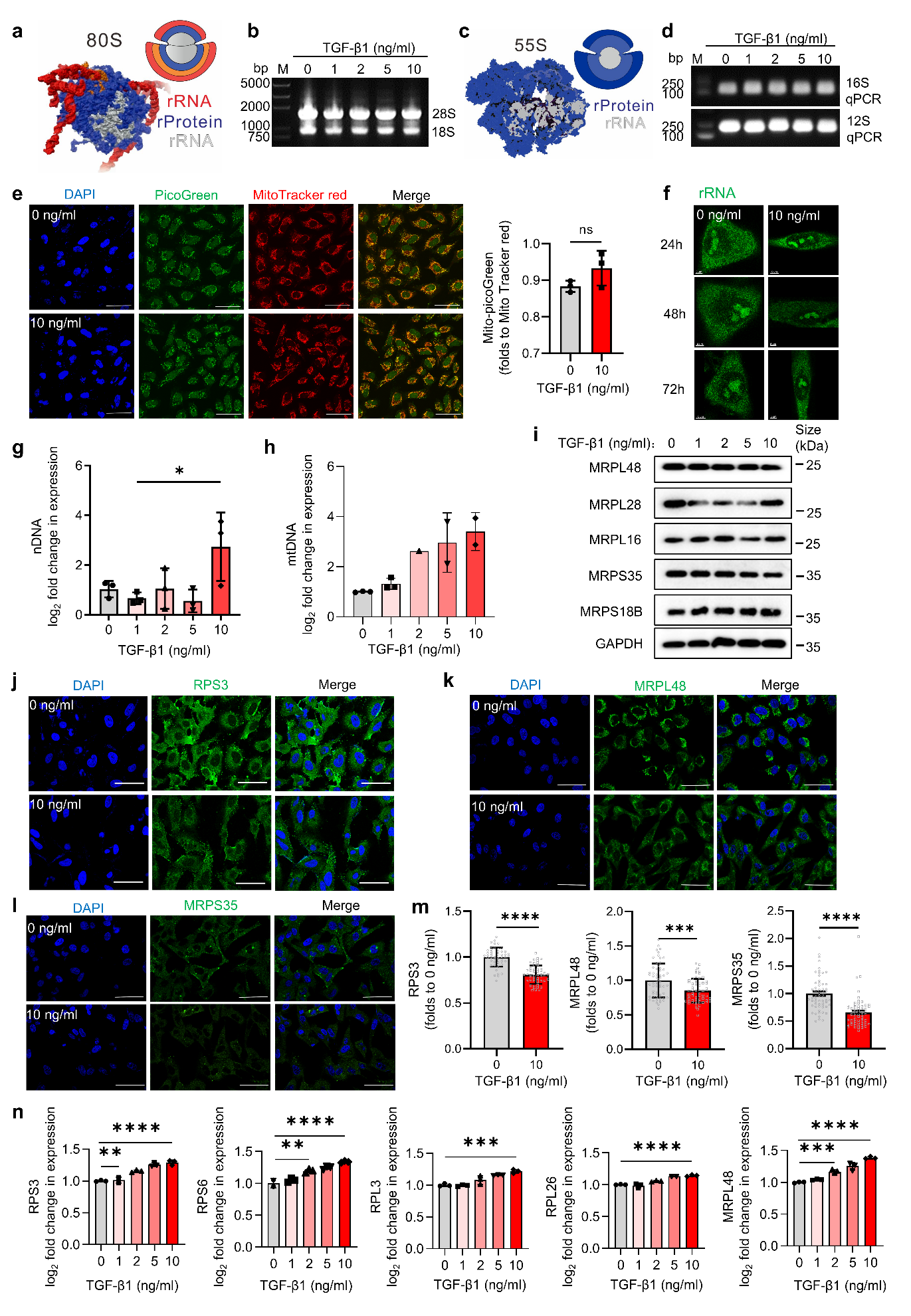


**Figure S9. TGF-β overload induced ribosome damage. (a)** The model of cytoplasmic ribosome in eukaryotic organisms. **(b)** Agarose gel electrophoresis of total RNA. **(c)** The model of mitochondrial ribosomes in eukaryotes. **(d)** Fluorescence quantitative PCR detection of mitochondrial 16S and, and their products were agarose gel electrophoresis. **(e)** Representative living cell image of mtDNA was detected by PicoGreen in A549 cells treated with different TGF-β1 concentrations, The images were taken using OLYMPUS FV3000 with a 60x oil-immersion objective under the same parameter settings, scale bar = 50 µm. The mean fluorescence intensity was obtained by Image J for statistics (*n* = 3). **(f)** The expression of rRNA in cells was detected by immunofluorescence. The images were taken using Nikon STED with a 100x oil-immersion objective under the same parameter settings. **(g, h)** The copy number of nDNA, mtDNA in cells was detected by QPCR (*n* = 3). **(i)** Immunoblot for protein expression levels of total MRPL48, MRPL28, MRPL16, MRPS35, MRPS18B and GAPDH in A549 lysates with different treatment conditions. **(j-m)** The expression of RPS3 (*n* = 50), MRPL48 (*n* = 60) and MRPS35 (*n* = 60) in A549 cells was detected by immunohistochemistry. The images were taken using OLYMPUS FV3000 with a 60x oil-immersion objective under the same parameter settings, scale bar = 50 µm. the mean fluorescence intensity was obtained by Image J for statistics. **(n)** The expression of RPS3, RPS6, RPL3, RPL26 and MRPL48 in cells was detected by qPCR (*n* = 3). One-way ANOVA with Tukey’s multiple comparisons test was performed. **P*<0.05; *****P*<0.0001.


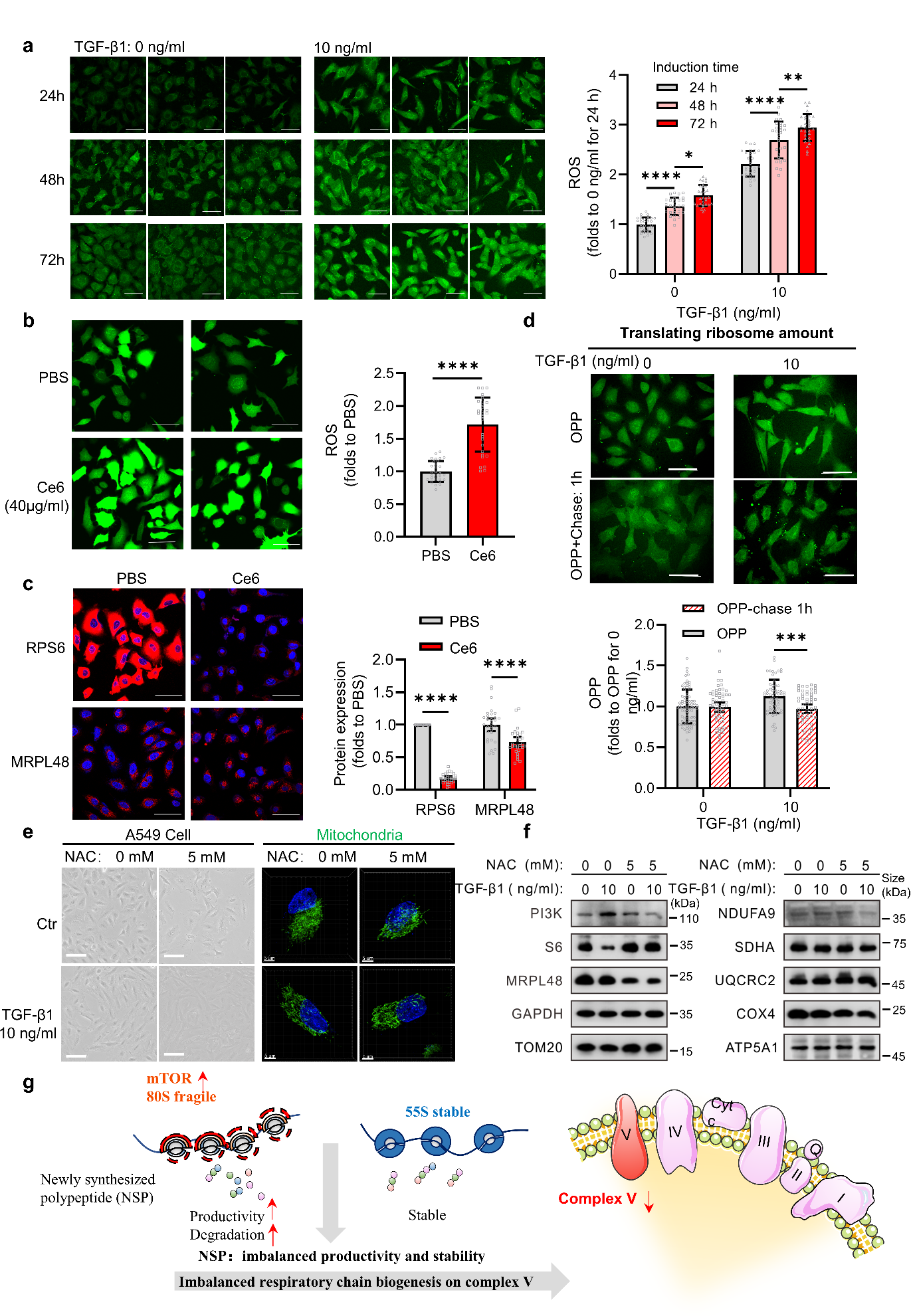


**Figure S10. Imbalanced tolerance of cyto- and mito-ribosomes to ROS stress. (a)** Changes of ROS after induction of TGF-β1 for 24h, 48h and 72h, respectively. The images were taken using OLYMPUS FV3000 with a 60x oil-immersion objective under the same parameter settings, scale bar = 50 µm. Fold changes of ROS in A549 cells, the mean fluorescence intensity was obtained by Image J for statistics (*n* = 30). **(b)** Changes of ROS after induction of TGF-β1 for PBS and Ce6, respectively. The images were taken using OLYMPUS FV1000 with a 60x oil-immersion objective under the same parameter settings, scale bar = 50 µm. Fold changes of ROS in A549 cells, the mean fluorescence intensity was obtained by Image J for statistics (*n* = 30). **(c)** The expression of RPS6 and MRPL48 was detected by immunofluorescence after PBS and Ce6 treatment, the images were taken using OLYMPUS FV3000 with a 60x oil-immersion objective under the same parameter settings, scale bar = 50 µm. Fold changes of RPS6 and MRPL48 expression in A549 cells after PBS and Ce6 treatment, the mean fluorescence intensity was obtained by Image J for statistics (*n* = 30). **(d)** Detection of protein stability in cytotranslational system by OPP pulse-chase experiments, the images were taken using OLYMPUS FV1000 with a 60x oil-immersion objective under the same parameter settings, scale bar = 50 µm, the mean fluorescence intensity was obtained by Image J for statistics (*n* = 60). **(e)** A549 was treated with TGF-β1 (10ng/ml), then NAC (ROS scavenger) was added, the morphology of cells was observed by DIC images under different treatment conditions, the images were taken using Nikon Ts2 inverted microscope, scale bar = 100 µm, the morphology of mitochondria were observed by OMX mode of structural light illumination microscope, scale bar = 5 µm. **(f)** Immunoblot for protein expression levels of total NDUFA9, SDHA, UQCRC2, COX4, ATP5a, TOM20, PI3K, RPS6, MRPL48, MRPL18 and GAPDH in A549 lysates with different treatment conditions. **(g)** Schematic diagram of Imbalanced cyto- and mito-translation products for respiratory chain complex subunits. Significance was determined using t test or One-way ANOVA with Tukey’s multiple comparisons test. ns: not significant; **P*<0.05; ***P*<0.01; ****P*<0.001; *****P*<0.0001.


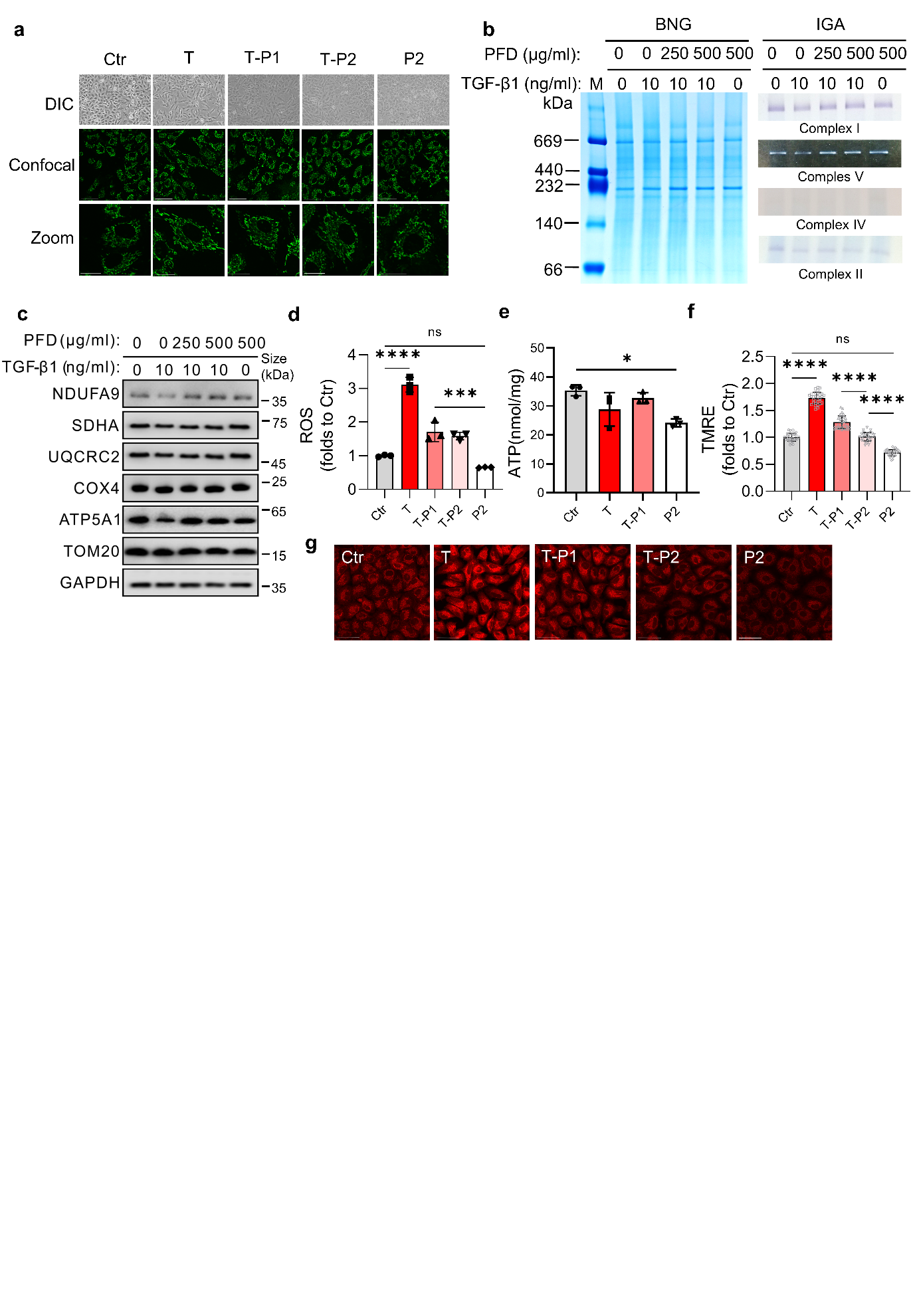


**Figure S11. Pirfenidone (PFD) prevents the progression of TGF-β induced pulmonary fibrosis. (a)** A549 cells were treated with different treatment conditions for 48 hours, The images were taken using Inverted fluorescence microscope and representative confocal microscopy. Different treatment conditions: T = TGF-β1 5 ng/ml, P1 = PFD 250 μg/ml, P2 = PFD 500 μg/ml. DIC images show the morphologic changes of cells under different treatment conditions, the images were taken using Nikon Ts2 inverted microscope, scale bar = 100 µm. The confocal images showed the changes of mitochondrial (green) morphology under different treatment conditions, the images were taken using OLYMPUS FV1000 with a 60x oil-immersion objective, scale bar = 50 µm, Zoom: scale bar = 20 µm. **(b)** In gel activity (IGA) for activity of mitochondrial respiratory complex I, II, IV and V in purified mitochondrial protein. **(c)** Immunoblot for protein expression levels of NDUFA9, SDHA, UQCRC2, COX4, ATP5a, TOM20, β-actin and GAPDH in A549 lysates with different treatment conditions. All lanes were loaded with the same amount of total protein. **(d)** Fold changes of ROS in A549 cells with different treatment conditions. The mean fluorescence intensity of DCF in A549 cells via flow cytometry (*n* = 3). **(e)** ATP content in A549 cells with different treatment conditions (*n* = 3). **(f)** Fold changes of TMRE in A549 cells with different treatment conditions, the mean fluorescence intensity was obtained by Image J for statistics (*n* = 30). **(g)** Mitochondrial membrane potential was detected by TMRE in A549 cells treated with different treatment conditions. Representative living cell images were taken using OLYMPUS FV3000 with a 60x oil-immersion objective under the same parameter settings, scale bar = 50 µm. One-way ANOVA with Tukey’s multiple comparisons test was performed. ns: not significant; ***P*<0.01; ****P*<0.001; *****P*<0.0001.

**Supplementary tables**

| **Table S1. Clinical characteristics and respiratory chain complex activity of the enrolled people.** | | | |
| --- | --- | --- | --- |
| Features | IPF | CTD-PF | HCs |
|  | N=9 | N=41 | N=9 |
| Age, years | 68.00 [59.00-70.00] | 58.00 [52.00-64.00] | 59.00 [46.0,68.00] |
| Gender, women/men | 4/5 | 24/16 * | 6/3 |
| Expiratory dyspnea | 6/9 | 6/41*** | NA |
| Dry cough | 7/9 | 9/41 *** | NA |
| FVC pre% | 69.55 [60.33,73.55] | 76.80 [62.30,92.10] | NA |
| DLCO pre% | 46.45 [37.55,57.70] | 47.40 [32.64,62.00] | NA |
| Complex I activity | 14.31 [10.65,24.02] | 14.57 [8.34,23.68] | 20.10 [13.10-37.99] |
| Complex II activity | 3.33 [1.79,5.42] | 3.41 [1.41,5.20] | 2.98 [1.48-5.52] |
| Complex III activity | 1.31 [0.41,2.48] | 0.99 [0.47,1.47] | 1.24 [0.60-2.77] |
| Complex IV activity | 0.98 [0.37,3.87] | 1.03 [0.46,4.68] | 1.07 [0.77-7.80] |
| Complex V activity | 0.10 [0.06,0.69] # | 0.25 [0.07,0.89] # | 1.09 [0.27-1.81] |
| Abbreviations: IPF, idopathic pulmonary fibrosis; CTD-PF, connective tissue disease associated pulonary fibrosis; HCs, health controls; HRCT, high resolution computerized tomography; FVC, forced vital capacity; DLco, diffusing capacity of the lung for carbon monoxide; | | | |
| Notes: No. positive/no. tested(%). Median [P25, P75] | | | |
| Statisical Significance * IPF group v.s. CTD-PF; # IPF group/ CTD-PF group v.s. HCs. **P <* 0.05, ***P <* 0.01*, ***P <* 0.001*.* | | |  |

**Table S2. Pathological semi-quantitative analysis score of mouse pulmonary fibrosis model.**

| Sample | | HE | | | | | | | | | Masson | | | |  | |
| --- | --- | --- | --- | --- | --- | --- | --- | --- | --- | --- | --- | --- | --- | --- | --- | --- |
|  |  | Pulmonary consolidation | | | Inflammatory infiltration | | | HE semi-quantitative analysis | | | Masson semiquantitative score | | | |  | |
| PBS1 | | 1+ | | | 1+ | | | 1+ | | | 1+ | | | |  | |
| PBS2 | | 1+ | | | 1+ | | | 1+ | | | 0 | | | |  | |
| PBS3 | | 1+ | | | 2+ | | | 2+ | | | 1+ | | | |  | |
| PBS4 | | 1+ | | | 1+ | | | 1+ | | | 1+ | | | |  | |
| PBS5 | | 1+ | | | 1+ | | | 1+ | | | 0 | | | |  | |
| BLM1 | | 4+ | | | 4+ | | | 4+ | | | 4+ | | | |  | |
| BLM2 | | 4+ | | | 4+ | | | 4+ | | | 4+ | | | |  | |
| BLM3 | | 4+ | | | 4+ | | | 4+ | | | 4+ | | | |  | |
| BLM4 | | 4+ | | | 4+ | | | 4+ | | | 4+ | | | |  | |
| BLM5 | | 4+ | | | 4+ | | | 4+ | | | 4+ | | | |  | |
| Notes: the semi-quantitative score of pathology is defined as: "no lesion" -; "mild lesion" 1+; "mild lesion" 2+; "moderate lesion" 3+; "severe lesion" 4+. | | | | | | | | | | | | | | |  | |
| **Table S3. Semi-quantitative analysis statistics of pathology.** | | | | | | | | | | | | | | |  | |
| Group | Number of animals | | HE | | | | | | | Masson | | | | | | |
|  |  |  | ━ | 1+ | | 2+ | 3+ | | 4+ | ━ | | 1+ | 2+ | 3+ | | 4+ |
| PBS | 5 | | / | 3/5 | | 2/5 | / | | / | 1/5 | | 2/5 | 2/5 | / | | / |
| BLM | 5 | | / | / | | / | / | | 5/5 | / | | / | / | / | | 5/5 |
| Note: a/b: a represents the number of animals with the degree of the lesion; b represents the total number of animals in this group. | | | | | | | | | | | | | | | | |

**Video S1. Mitochondrial dynamic changes of pulmonary epithelial cells induced by TGF-β.** The 3D video shows the changes of mitochondrial 3D morphology in A549 cells treated with different TGF-β1 concentrations for 48 hours. Time video shows the dynamic changes of mitochondria in 1 minute.
